# Comparing Different Diagnostic Guidelines for Gestational Diabetes Mellitus in Relation to Birthweight in Sri Lankan Women

**DOI:** 10.3389/fendo.2018.00682

**Published:** 2018-11-15

**Authors:** Thiran Dias, Shahul Hameed Mohamed Siraj, Izzuddin Mohamed Aris, Ling-Jun Li, Kok Hian Tan

**Affiliations:** ^1^Department of Obstetrics and Gynaecology, Faculty of Medicine, University of Colombo, Colombo, Sri Lanka; ^2^Department of Obstetrics and Gynaecology, Teaching Hospital, Batticaloa, Sri Lanka; ^3^Division of Chronic Disease Research Across the Lifecourse, Department of Population Medicine, Harvard Medical School and Harvard Pilgrim Health Care Institute, Boston, MA, United States; ^4^Division of O&G, KK Women's and Children's Hospital, Singapore, Singapore; ^5^O&G Academic Clinical Programme (ACP), Duke-NUS Medical School, Singapore, Singapore; ^6^Singapore Eye Research Institute, Singapore National Eye Centre, Singapore, Singapore

**Keywords:** gestational diabetes mellitus, screening, pregnancy, IADSPG, WHO 1999

## Abstract

**Introduction:** While the International Association of Diabetes and Pregnancy Study Groups (IADPSG) criteria is widely adopted in many countries, clinicians have questioned the applicability of these diagnostic thresholds for different races/ethnicities. We first compared the prevalence of gestational diabetes mellitus (GDM) diagnosed with different criteria including IADPSG, World Health Organization (WHO) 1999 and Sri Lankan national guidelines, and subsequently related individual guidelines-specific GDM prevalence to offspring birthweight in Sri Lanka.

**Materials and Methods:** We retrospectively collected data on singleton pregnancies (*n* = 795) from two tertiary hospitals in Sri Lanka. We applied three diagnostic guidelines to define GDM, namely IADPSG criteria, the Sri Lankan national and WHO 1999 guidelines. We calculated the age- and first booking BMI-adjusted prevalence rates of GDM and assessed the association of GDM (using each guideline) with birthweight.

**Results:** The age- and first booking BMI-adjusted GDM prevalence rates were 31.2, 28.0, and 13.1% for IADPSG criteria, Sri Lankan national and WHO 1999 guidelines, respectively. The IADPSG criteria identified 90 distinctive GDM cases at a lower cut-off of fasting glucose (from 5.1 to 5.5 mmol/L) while Sri Lankan national guideline identified 15 distinctive GDM cases at a lower cut-off for 2-h glucose (from 7.8 to 8.4 mmol/L). After adjusting for age, GDM diagnosed by IADPSG criteria was associated with higher birthweight [90.8 g, 95% CI: 10.8, 170.9], while the associations for GDM diagnosed either by Sri Lankan national or WHO 1999 guidelines were not significant.

**Conclusion:** Adopting the IADPSG criteria for diagnosing GDM may be important in Sri Lankan pregnant population.

## Introduction

Gestational diabetes mellitus (GDM) is a condition of glucose intolerance first recognized during pregnancy (1). Currently, the guidelines for diagnosing GDM, as recommended by the International Association of Diabetes and Pregnancy Study Groups (IADPSG) and adopted by the World Health Organization (WHO) in 2013 (2), include an elevation in either fasting (≥5.1 mmol/L), 1-h (≥10.0 mmol/L), or 2-h (≥8.5 mmol/L) venous plasma glucose level after a 75-gram glucose intake (1). These thresholds are based on the average glucose values at which odds for birthweight >90th percentile, cord C-peptide >90th percentile, and percent body fat >90th percentile reached 1.75 times the estimated odds of these outcomes at mean glucose values, based on fully adjusted logistic regression models (3).

While the IADPSG criteria is widely adopted in many countries, clinicians have questioned the applicability of these diagnostic thresholds for different ethnicities (4–6). Recent findings from a Danish cohort demonstrated that the IADPSG criteria threshold for fasting glucose (≥5.1 mmol/L) appeared inappropriate for Denmark (6). The study reported that the IADPSG criteria had classified an unmanageable number of women as having GDM who were actually at low risk of pregnancy complications; and subsequently diverted finite health care resources from other areas.

The 10-year incidence of developing T2D among GDM mothers (vs. non-GDM mothers) is as high as 7- to 10-folds (7, 8). Even though the prevalence of GDM and Type 2 diabetes (T2D) is known to be historically high in South Asian populations (9–15), none of these studies have addressed the applicability of IADPSG criteria in South Asian populations compared with other diagnostic guidelines, together with its relationship with any birth outcomes. Therefore, it would be important to test the new GDM diagnostic guidelines in a region where the burden of hyperglycaemia is critical (13). In this study, we aimed to examine: (1) The prevalence of GDM diagnosed by different guidelines including WHO 1999 (16), IADPSG criteria and the Sri Lankan national guidelines (17); (2) The association between guidelines-specific GDM prevalence and offspring birthweight, in a hospital-based observational study among Sri Lankan pregnant women.

## Methods

This is a retrospective longitudinal and hospital-based study. Between January 2016 and January 2018, clinicians reviewed the medical records of 795 women during their pregnancy and at delivery. All patients attended two tertiary hospitals with obstetric care in Sri Lanka, namely Colombo North Teaching Hospital (CNTH) (*n* = 543) and Batticaloa Teaching Hospital (BTH) (*n* = 252).This study utilized de-identified data from medical records, and is therefore exempted from ethical approval per regulations governing research with human subjects. A final number of 795 women with singleton pregnancies without pre-existing T2D were included in the study analysis and had the following variables: age, height and weight at first booking visit (≤13 weeks of gestation) (ZT-120 Health Scale, Digital Medical & Health-Care Scales, Hong Kong, China), gestational age at first booking, 75 g oral glucose tolerance test (OGTT) with fasting, 1- and 2-h glucose readings after 20 weeks of gestation (Mindray BS-800, Chemistry Analyzer, Shanghai, China), and offspring birthweight. We calculated body mass index (BMI) at the first booking visit (as an estimate of pre-pregnancy BMI) as weight in kilograms over square of height in meters (18).

We applied three diagnostic thresholds in our study to define GDM: (1) IADPSG criteria: fasting glucose ≥5.1 mmol/L and/or 1-h glucose ≥10.0 mmol/L and/or 2-h ≥8.5 mmol/L; (2) Sri Lankan national guidelines: fasting glucose ≥5.6 mmol/L and/or 1-h glucose ≥10.0 mmol/L and/or 2-h ≥7.8 mmol/L (17); (3) WHO 1999: fasting glucose ≥7.0 mmol/L and/or 2-h ≥7.8 mmol/L (16).

We first calculated crude GDM prevalence, and subsequently used logistic regression to calculate adjusted GDM prevalence rates by including age and first booking BMI as independent variables in the model. The sum of individual GDM prevalence estimate represented the age- and first booking BMI-adjusted GDM prevalence in this study. We also studied the association between GDM and birthweight in unadjusted, age-adjusted, and age-/first booking BMI-adjusted models. Sensitivity analysis included infant sex and gestational age at GDM diagnosis. We conducted all statistical analyses using STATA (version 14.0, STATA corp, Texas, US). We used Venn diagrams to represent the individual and overlapping diagnosis of GDM by each time point of glucose level.

## Results

The mean and standard deviation (SD) of maternal age, first booking BMI and offspring birthweight was 27.9 years (5.7), 23.9 kg/m^2^ (5.0), and 3030.0 g (508.7), respectively. Since women from BTH were younger and had higher first booking BMI, fasting, and 1 h glucose levels than women in NCTH (Table 1), we further calculated crude and age- and first booking BMI-adjusted GDM prevalence for comparison. We found significant differences in crude and age- and first booking BMI-adjusted GDM prevalence using Sri Lankan national guidelines (28.0 vs. 27.2%, *p* < 0.01), but not in IADPSG (31.2 vs. 30.8%, *p* = 0.07) and WHO 1999 guidelines (13.1 vs. 13.3%, *p* = 0.12).

**Table 1:** Characteristics and GDM prevalence in two Sri Lanka tertiary hospitals.

	**Total *n* = 795 mean, SD**	**CNTH *n* = 543 mean, SD**	**BTH *n* = 252 mean, SD**	***p*-value**
**MATERNAL CHARACTERISTICS**				
Age, years	27.9, 5.7	28.8, 5.4	25.9, 5.9	<0.001
BMI at first booking, kg/m^2^	23.9, 5.0	23.4, 4.8	24.9, 5.4	<0.001
OGTT, Fasting mmol/L	4.7, 0.9	4.6, 0.8	4.9, 1.0	0.002
OGTT, 1-h mmol/L	7.5, 2.0	7.4, 1.9	7.9, 2.2	0.003
OGTT, 2-h mmol/L	6.1, 1.6	6.0, 1.6	6.2, 1.6	0.09
**FETAL CHARACTERISTICS**				
Gender, Male	373 (46.9%)	264 (48.6%)	109 (43.3%)	<0.001
Birthweight, g	3030.0, 508.7	3006.0, 542.9	3075.5, 433.9	0.06
**MATERNAL GDM PREVALENCE (≥20 WEEKS GESTATION) BY IADPSG**				
Crude	248 (31.2%)	148 (27.3%)	100 (39.7%)	<0.001
Age and first-booking BMI adjusted	31.2%	30.8%	32.2%	0.07
**MATERNAL GDM PREVALENCE (≥20 WEEKS GESTATION) BY WHO 1999**				
Crude	104 (13.1%)	64 (11.8%)	40 (15.9%)	<0.001
Age and first-booking BMI adjusted	13.1%	13.3%	12.6%	0.12
**MATERNAL GDM PREVALENCE (≥20 WEEKS GESTATION) BY SRI LANKA NATIONAL GUIDELINE**				
Crude	173 (21.8%)	102 (18.8%)	71 (28.2%)	<0.001
Age and first-booking BMI adjusted	28.0%	27.2%	29.7%	<0.01

*CNTH, Colombo North Teaching Hospital; BTH, Batticaloa Teaching Hospital; OGTT, oral glucose tolerance test; SD, standard deviation; BMI, body mass index*.

*p-value done by either student's t-test or χ^2^ test*.

In unadjusted models, GDM diagnosed by IADPSG criteria or Sri Lankan national guidelines were significantly associated with birthweight [IADPSG: β 100.1 g, 95% confidence interval [CI] (20.7, 179.6); Sri Lanka: 107.5 g (6.7, 208.2)]. After adjusting for age, the association between GDM diagnosed by IADPSG criteria with birthweight remained significant [90.8 g (10.8, 170.9)], while the associations for GDM diagnosed by Sri Lankan national guidelines attenuated to non-significance [100.2 g (−0.9, 201.4)]. After adjusting for age and first booking BMI, the associations were attenuated both in IADPSG criteria [35.3 g (−45.0, 115.6)] and Sri Lankan national guidelines [33.3 g (−69.1, 135.8)]. Linear regression of GDM diagnosed by WHO 1999 was not associated with birthweight in either unadjusted or adjusted models (Supplementary Table 1).

**Figure 1:**
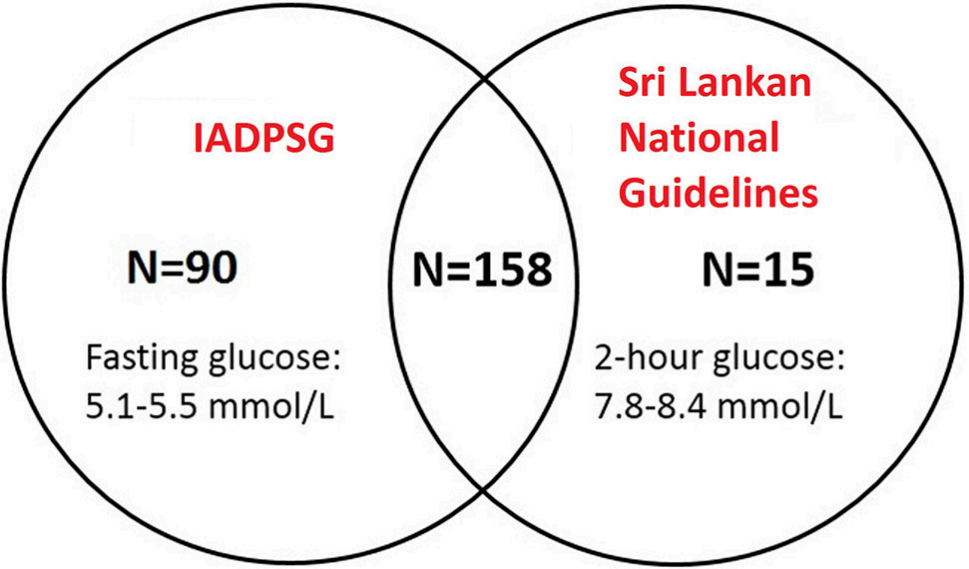
The first two top circles showed the overlapping diagnosis of GDM by using IADPSG and Sri Lankan national guidelines.

The IADPSG criteria thresholds identified 90 distinctive GDM cases at a lower cut-off of fasting glucose criteria (from 5.1 to 5.5 mmol/L) while Sri Lankan national guidelines identified 15 distinctive GDM cases at a lower cut-off 2-h glucose criteria (from 7.8 to 8.4 mmol/L) (Figure 1). Fasting glucose had the strongest associations with birthweight compared with 1 and 2-h glucose level (Supplementary Table 2). The adjusted estimates of birthweight in relation to GDM diagnosis did not seem to differ greatly, either by IADPSG alone [37.6 g (−78.2, 153.4)] or by both IADPSG and Sri Lankan national guidelines [29.6 g (−66.0, 125.3)] (Supplementary Table 3). The additional adjustment of baby sex and gestational age at GDM diagnosis did not change our observation significantly.

## Discussion

Our findings showed that in a representative group of women with singleton pregnancies from two of Sri Lanka's reputable tertiary hospitals, using IADPSG criteria identified more GDM cases among women with lower fasting glucose levels, and its association with birthweight is more significant than both Sri Lankan national and WHO 1999 guidelines. Adopting the IADPSG criteria for diagnosing GDM might be important in Sri Lankan pregnant women.

Clinicians have questioned whether a single diagnostic criterion is applicable for pregnant women of different races and ethnicities (4–6). Although the HAPO study concluded that associations between gestational glycemia and pregnancy outcomes were generalizable across different countries (1), recent findings from a Danish cohort have challenged these conclusions (6). The authors found no evidence of excessive fetal growth or hypertensive disorders of pregnancy (HDP) in women with untreated GDM, and concluded that using fasting glucose ≥5.1 mmol/L is an inappropriate and cost-ineffective approach for GDM diagnosis in Danish pregnant women (6). Three prior studies conducted in Indian pregnant women had shown that IADPSG picked up more GDM than WHO 1999 guidelines, and IADPSG-defined GDM was associated with lower maternal socio-economic status and more adverse feto-maternal outcomes (11, 12, 14). To our knowledge, no study has compared the relationship of different GDM diagnostic guidelines with birth outcomes in South Asian populations, where the prevalence of hyperglycaemia during pregnancy is higher than other Asian ethnicities (13).

In our study of Sri Lankan pregnant women, we found GDM diagnosed using the IADPSG criteria, or using Sri Lankan guidelines, had significant associations with birthweight in unadjusted models, yet the associations attenuated to non-significance after adjusting for age and first booking BMI. It is notable that effect estimates for birthweight did not differ greatly among women diagnosed with GDM by IADPSG only and in those diagnosed with GDM by IADPSG and Sri Lankan guidelines. Furthermore, the IADPSG criteria appeared to have a better diagnostic value among all three guidelines, in terms of identifying cases of GDM and in predicting birthweight. Taken together, our results suggest that adopting the IADPSG criteria for diagnosing GDM may be more clinically practical Sri Lankan women.

The strengths of our study include a relatively large sample of pregnant women, complete glucose profile with OGTT at fasting, 1 and 2-h time points, weight and height measured at booking according to standard operation procedures (SOPs) Furthermore, our data are important in terms of representation from tertiary care institutions of Sri Lanka by comparing various GDM diagnostic criteria, where hyperglycaemia during pregnancy is historically high. However, our study is not without limitations. Due to the logistical difficulties, we were unable to retrieve data on parity, GDM treatment during pregnancy, family history and other maternal determinants, which prevented us from observing the fully adjusted estimates in our association of interest. In addition, we did not have either enough large sample to compare the association between different guidelines and rare pregnancy outcomes (e.g., pre-eclampsia, macrosomia, still birth), or long enough follow-up data to observe childhood obesity, which also prevented us from further differentiating the clinically indicative values of the three GDM diagnostic guidelines. Lastly, as this study uses retrospectively-collected data from medical records, there may be potential for errors in documentation. Furthermore, our selection of exposure may not represent the general GDM prevalence in Sri Lankan women.

In summary, the IADPSG criteria identified more cases of GDM which were associated with greater birthweight in Sri Lankan pregnant population, compared with its national GDM guidelines. Our findings provide additional insights regarding applicability and clinical implication of IADPSG criteria in South Asian pregnant populations. Further studies in a larger population with a longer follow-up period are warranted, before implementation of any changes to current guidelines for diagnosing GDM in Sri Lankan women.

## Ethics statement

All procedures followed were in accordance with the ethical standards of the responsible committee on human experimentation (institutional and national) and with the Helsinki Declaration of 1975, as revised in 2008. This study was conducted retrospectively on existing medical record, which has been approved by local IRB committee. No informed consent was required from all patients included in the study.

## Author contributions

TD and SS conducted the study and edited the manuscript. IA reviewed and edited the manuscript. L-JL designed the study, performed data analyses, and wrote the manuscript. KT designed the study, reviewed, and edited the manuscript. All authors have provided their final approval of the version to be submitted to Frontiers in Endocrinology. L-JL is responsible for the integrity of the work as a whole.

## Conflict of interest statement

The authors declare that the research was conducted in the absence of any commercial or financial relationships that could be construed as a potential conflict of interest.
