# Supplementary material for: Comparing Different Diagnostic Guidelines for Gestational Diabetes Mellitus in Relation to Birthweight in Sri Lankan Women
Source: Front Endocrinol (Lausanne). 2018 Nov 15;9:682. doi: 10.3389/fendo.2018.00682 (PMC6262349; doi:10.3389/fendo.2018.00682)
Supplement: Supplementary file 1 [file Table_1.DOCX]

**Supplementary Table 1. Prediction value of GDM with birthweight by different diagnostic approaches**

|  | Birthweight, g | |
| --- | --- | --- |
|  | β (95% CI) | p value |
| ***IADPSG GDM diagnosis*** | | |
| Unadjusted | 100.1 (20.7, 179.6) | 0.01 |
| Adjusted for age | 90.8 (10.8, 170.9) | 0.03 |
| Adjusted for age and first booking BMI | 35.3 (-45.0, 115.6) | 0.39 |
| ***Sri Lanka national modified guideline GDM*** | | |
| Unadjusted | 107.5 (6.7, 208.2) | 0.04 |
| Adjusted for age | 100.2 (-0.9, 201.4) | 0.05 |
| Adjusted for age and first booking BMI | 33.3 (-69.1, 135.8) | 0.52 |
| ***WHO 1999*** | | |
| Unadjusted | 48.0 (-60.9, 156.9) | 0.39 |
| Adjusted for age | 28.1 (-82.5, 138.7) | 0.62 |
| Adjusted for age and first booking BMI | -16.8 (-125.5, 91.9) | 0.76 |
