# Supplementary material for: Comparing Different Diagnostic Guidelines for Gestational Diabetes Mellitus in Relation to Birthweight in Sri Lankan Women
Source: Front Endocrinol (Lausanne). 2018 Nov 15;9:682. doi: 10.3389/fendo.2018.00682 (PMC6262349; doi:10.3389/fendo.2018.00682)
Supplement: Supplementary file 2 [file Table_2.DOCX]

**Supplementary Table 2. Prediction value of OGTT results with birthweight**

|  | Birth weight, g | |
| --- | --- | --- |
|  | β (95% CI) | p value |
| ***Fasting glucose, each 1 mmol/L increase*** | | |
| Unadjusted | 54.3 (13.7, 94.8) | <0.01 |
| Adjusted for age | 48.7 (7.7, 89.7) | 0.02 |
| Adjusted for age and first booking BMI | 29.2 (-11.4, 69.8) | 0.16 |
| ***Fasting glucose z-score, each 1 SD increase (SD 0.89 mmol/L)*** | | |
| Unadjusted | 48.1 (12.2, 84.1) | <0.01 |
| Adjusted for age | 43.2 (6.8, 79.6) | 0.02 |
| Adjusted for age and first booking BMI | 25.9 (-10.1, 61.9) | 0.16 |
| ***One-hour glucose, each 1 mmol/L increase*** | | |
| Unadjusted | 14.5 (-3.9, 32.9) | 0.12 |
| Adjusted for age | 10.8 (-8.1, 29.7) | 0.26 |
| Adjusted for age and first booking BMI | -0.1 (-18.8, 18.7) | 0.99 |
| ***One-hour glucose z-score, each 1 SD increase (SD 2.02 mmol/L)*** | | |
| Unadjusted | 29.3 (-8.0, 66.6) | 0.12 |
| Adjusted for age | 21.8 (-16.4, 60.0) | 0.26 |
| Adjusted for age and first booking BMI | -0.2 (-38.1, 37.7) | 0.99 |
| ***Two-hour glucose, each 1 mmol/L increase*** | | |
| Unadjusted | 12.0 (-11.3, 35.3) | 0.31 |
| Adjusted for age | 7.3 (-16.5, 31.0) | 0.55 |
| Adjusted for age and first booking BMI | -1.1 (-24.5, 22.2) | 0.93 |
| ***Two-hour glucose z-score, each 1 SD increase (SD 1.57 mmol/L)*** | | |
| Unadjusted | 18.9 (-17.8, 55.5) | 0.31 |
| Adjusted for age | 11.4 (-26.0, 48.8) | 0.55 |
| Adjusted for age and first booking BMI | -1.8 (-38.5, 35.0) | 0.93 |
