# Supplementary material for: Comparing Different Diagnostic Guidelines for Gestational Diabetes Mellitus in Relation to Birthweight in Sri Lankan Women
Source: Front Endocrinol (Lausanne). 2018 Nov 15;9:682. doi: 10.3389/fendo.2018.00682 (PMC6262349; doi:10.3389/fendo.2018.00682)
Supplement: Supplementary file 3 [file Table_3.docx]

**Supplementary Table 3. Prediction value of GDM with IADPSG and Sri Lanka national guidelines with birthweight**

| GDM diagnostic guidelines | Birthweight, g | | |
| --- | --- | --- | --- |
|  | Unadjusted  β (95% CI)  p value | Adjusted for age  β (95% CI)  p value | Adjusted for age and first booking BMI  β (95% CI)  p value |
| Non-GDM both by IADPSG and Sri Lanka national guidelines (n=532) | Reference | Reference | Reference |
| GDM by both IADPSG and Sri Lanka national guideline (n=158) | 105.3 (10.9, 199.7)  p=0.03 | 91.4 (-4.2, 187.1)  p=0.06 | 29.6 (-66.0, 125.3)  p=0.54 |
| GDM only by Sri Lanka national guideline (n=15) | -102.2 (-382.1, 177.8)  p=0.47 | -98.9 (-378.5, 180.7)  p=0.49 | -103.8 (-377.1, 169.5)  p=0.46 |
| GDM only by IADPSG guideline (n=90) | 84.0 (-33.6, 201.7)  p=0.16 | 83.2 (-34.3, 200.7)  p=0.17 | 37.6 (-78.2, 153.4)  p=0.52 |
